# The Use of Neurologic Music Therapy in Post-Stroke Aphasia Recovery: A Case Report on Linguistic Improvements and fMRI Correlates

**DOI:** 10.3390/jcm14103436

**Published:** 2025-05-14

**Authors:** Federica Impellizzeri, Maria Grazia Maggio, Lilla Bonanno, Michael Thaut, Corene Hurt, Angelo Quartarone, Rocco Salvatore Calabrò

**Affiliations:** 1 IRCCS Centro Neurolesi Bonino Pulejo, SS 113 C.da Casazza, 98124 Messina, Italy; federica.impellizzeri@irccsme.it (F.I.); mariagrazia.maggio@irccsme.it (M.G.M.); angelo.quartarone@irccsme.it (A.Q.); roccos.calabro@irccsme.it (R.S.C.); 2 Music and Health Science Research Collaboratory, Faculty of Music, University of Toronto, Toronto, ON M5S 1C5, Canada; michael.thaut@utoronto.ca (M.T.); corene.thaut@utoronto.ca (C.H.)

**Keywords:** post-stroke aphasia, neurorehabilitation, melodic intonation therapy, neurologic music therapy, functional neuroimaging, fMRI

## Abstract

**Background and Objectives:** Post-stroke aphasia (PSA) severely limits communication and quality of life. This case study explores the impact of an integrated Neurologic Music Therapy (NMT) approach, combining Melodic Intonation Therapy (MIT) and Therapeutic Singing (TS), on language recovery and brain reorganization in a 59-year-old woman with non-fluent motor aphasia following an ischemic stroke. **Methods:** Over 8 weeks, the patient underwent 24 sessions of MIT alongside standard speech therapy. Language abilities were assessed using the Western Aphasia Battery-Revised, while fMRI scans captured neurophysiological changes pre- and post-intervention. **Results:** The results showed significant language improvements: spontaneous speech increased by 68.9%, auditory comprehension by 30.8%, and naming by 83.3%. The Aphasia Quotient rose from 39.3 to 61.4, marking a shift from severe to moderate aphasia. Neuroimaging revealed heightened activation in both hemispheres, especially in the superior frontal and parietal regions, supplementary motor area, and superior temporal gyrus. Increased engagement of the limbic system, particularly the paracingulate gyrus, pointed to emotional involvement and widespread cortical reorganization. **Conclusions:** These findings highlight the effectiveness of integrating MIT and TS with emotionally meaningful music, supporting language recovery and neural plasticity in PSA.

## 1. Introduction

Stroke is the leading cause of aphasia, affecting 20–40% of individuals acutely and persisting into the chronic phase for up to 25% of survivors, profoundly disrupting the ability to communicate and significantly diminishing quality of life and social participation [1,2,3,4,5,6]. Post-stroke aphasia (PSA) impacts both expressive and receptive language skills and is frequently linked with mood disorders [7,8,9]. Broca’s aphasia, a type of PSA, features expressive agrammatism and morphosyntactic challenges, primarily affecting the left inferior frontal regions—particularly the pars triangularis and pars opercularis, crucial for syntactic processing [10,11,12,13,14,15]. Managing PSA requires a multidisciplinary approach tailored to individual clinical profiles, including symptoms, lesion locations, and the degree of preserved cognitive and linguistic functions [16,17]. Since the 1970s, Melodic Intonation Therapy (MIT), now encompassed within Neurologic Music Therapy (NMT), has proven effective for rehabilitating individuals with non-fluent aphasia. This approach leverages the melodic and rhythmic elements of music to enhance speech production, demonstrating significant therapeutic benefits [18,19,20,21,22,23,24,25]. The most commonly adopted MIT protocol in the literature is structured to include three sessions per week over a two-month period [26,27]. This dosage appears to promote functional improvements in cases of chronic non-fluent aphasia. Several studies have investigated the effects of MIT not only on language outcomes but also on functional brain organization [26,28,29,30,31,32,33,34,35,36]. For instance, fMRI, as a reliable and non-invasive tool that enables the detection of changes in brain activity with a high spatial resolution, is used even in single-case studies to track cortical reorganization in relation to behavioral interventions, offering complementary evidence to standardized language testing. Various authors have demonstrated increased activation in right-hemisphere homologs of Broca’s area and stronger auditory–motor connectivity following intensive MIT in individuals with chronic aphasia [27]. Recently, García-Casares et al. further reported BOLD signal changes after melodic-based training, highlighting the involvement of the frontotemporal and limbic regions [37]. These findings support the use of functional neuroimaging, particularly fMRI, to detect treatment-related neuroplasticity in language and emotion-related networks.

Moreover, studies have shown increasing patient engagement through familiar musical material. In fact, singing-based interventions, including choral singing and singing techniques of NMT like Therapeutic Singing (TS), can support language recovery in people with aphasia by activating bilateral frontotemporal networks, engaging the mirror neuron system, and promoting verbal fluency through entrainment mechanisms [33,38,39,40].

Despite advances in neuroimaging that have facilitated detailed studies on MIT, questions remain regarding its effectiveness, especially concerning the integration of personalized NMT interventions and comprehensive neurophysiological assessments [41,42].

This pilot study uniquely applies MIT and integrates TS to enhance not only speech production but also emotional engagement and motivation, combining a hierarchical structure-based NMT language technique with the singing of personalized, emotionally meaningful songs. Through comprehensive neuropsychological and neuroimaging assessments, this approach aims to reveals how tailored musical procedures could enhance neurorehabilitation in PSA patients, offering new insights into music-based interventions.

## 2. Materials and Methods

### 2.1. Case Description

This report follows the SCRIBE 2016 guidelines for single-case behavioral intervention studies. The patient was a 59-year-old Italian woman, right-handed, with no prior history of neurological or psychiatric disorders. She worked as a primary school teacher, was married, and had two adolescent children. Her educational background and former occupation were relevant for understanding her pre-stroke cognitive baseline and communicative needs. In September 2018, she experienced an acute ischemic stroke affecting the left Sylvian territory, with radiological evidence of infarction involving the inferior frontal gyrus and adjacent perisylvian areas. This lesion pattern was consistent with non-fluent aphasia (Broca’s aphasia), as confirmed by both clinical examination and neuroimaging. The patient was admitted to the Emergency Neurology and Stroke Unit at IRCCS Centro Neurolesi “Bonino-Pulejo” in Messina, one year after her initial ischemic stroke, and presented with severe agramatism, markedly reduced fluency, and phonological and articulatory deficits consistent with apraxia of speech. Motor examination revealed a mild right hemiparesis and spastic hypertonia of the right upper limb, which gradually improved over time.

The patient was enrolled in the clinical protocol following ethical approval and provided written informed consent. Selection was based on the presence of chronic non-fluent aphasia, with preserved comprehension and minimal spontaneous recovery, at the time of intervention. The patient’s musical background was evaluated using the Musical–Sound Anamnesis Form. She reported no formal training in singing or playing musical instruments. A baseline language evaluation was performed before starting the treatment program, and the inclusion criteria were confirmed through a multidisciplinary assessment conducted by a neurologist, a neuropsychologist, and a speech–language therapist. The inclusion criteria were a documented diagnosis of central nervous system pathology (confirmed via imaging), preserved auditory comprehension (Token Test ≥ 5; ENPA word comprehension ≥ 10), and the ability to provide informed consent. The exclusion criteria included severe auditory comprehension deficits (Token Test < 5; ENPA < 10), a history of epilepsy, significant psychiatric disorders, sensory hearing impairments, and any contraindications to MRI scanning. At the time of enrollment, the patient exhibited extreme communicative difficulties, requiring significant listener support. Her verbal output was effortful and fragmented, with severely impaired prosody and articulatory control. These impairments were functionally disabling, severely affecting her social interactions and emotional well-being. The detailed description of her baseline assessments, intervention plan (MIT and TS), and outcome measures is provided in the following sections.

### 2.2. Outcomes Measures

All assessments were conducted by a trained neuropsychologist and a certified speech–language pathologist, both independent from the therapy team. The evaluations took place in a quiet, dedicated testing room. A week before (baseline: T0) and after (T1) the intervention, the patient underwent evaluations, including the Western Aphasia Battery-Revised (WAB-R) [43], to determine the Aphasia Quotient (AQ) Index. The AQ is a weighted average of subtest scores related to verbal production—spontaneous speech, auditory–verbal comprehension, repetition, naming, and word finding—that quantifies linguistic ability. The AQ scores categorize the severity of aphasia as very severe (0–25), severe (26–50), moderate (51–75), or mild (76+). Additionally, the Token Test [44] assesses comprehension and ideomotor skills, and the Aphasic Depression Rating Scale (ADRS) [45] evaluates emotional health, accounting for aphasic individuals’ communication challenges.

### 2.3. Procedures

The study protocol was approved by the Ethical Committee of IRCCS Centro Neurolesi “Bonino-Pulejo”, Messina, Italy (approval No. 16/2019, June 2019), and was conducted in accordance with the ethical standards laid down in the 1964 Declaration of Helsinki and its later amendments. The patient’s capacity to provide informed consent was assessed by the clinical team based on her cognitive and communicative functioning. A neuropsychological examination confirmed that she was alert, fully oriented, and capable of understanding the purpose and procedures of the intervention. As such, she personally signed the informed consent form. Her family was informed and remained involved throughout the rehabilitation process, offering practical and emotional support.

This intervention was structured according to NMT principles and followed a single-case design consistent with SCRIBE 2016 recommendations. Prior to initiating the intervention, a Musical–Sound Anamnesis Form was administered to the patient’s caregivers to gather information on her musical preferences, personal history with music, and emotionally salient songs. This step was used to tailor part of the intervention and the fMRI auditory stimuli to emotionally meaningful content.

The treatment plan was structured to integrate into the traditional speech therapy program both NMT techniques, specifically MIT and TS. After giving informed consent to this study protocol, the patient participated in 24 sessions, scheduled three times per week over the course of eight weeks, corresponding to the standard duration of inpatient rehabilitation at our facility. Each session lasted approximately 45 min of MIT and TS, in addition to conventional speech therapy sessions scheduled separately. Each MIT-TS session was divided into two phases.

The first 25 min of each session were dedicated to MIT. These exercises were based on melodic intonation, rhythmic pacing, and a hierarchical progression of phrase complexity. The MIT intervention followed a structured NMT protocol delivered by a certified Neurologic Music Therapist and composed of four progressive levels, each subdivided into five steps, moving from unison singing with tapping to independent speech with natural prosody. Syllable-level tapping was selected to provide precise motor–rhythmic cueing, facilitating articulation, segmentation, and speech timing. A set of functional, high-frequency phrases relevant to daily communication was used. Examples include “Mi chiamo…”, “Come stai?”, “Ho fame”, “Ti voglio bene”, “Accendi la luce”, and “Andiamo in palestra”. Progression was monitored using a therapist scoring sheet based on accuracy and level completion. Each 25 min MIT session began with a review of previously mastered phrases, followed by the introduction of a new phrase when performance allowed. The therapist applied the structured hierarchy described in the Supplementary Materials File, but the number of phrases addressed per session varied depending on the patient’s responsiveness, fatigue, and cognitive status. On some days, a single phrase was practiced throughout; on others, more than one phrase could be introduced. The intervention was adapted in real-time by the certified NMT therapist, ensuring both therapeutic intensity and patient-centered flexibility. No additional practice was conducted between sessions, as the intervention was delivered entirely within the inpatient rehabilitation program by the NMT-certified therapist.

The remaining 20 min of each session were allocated to TS, during which the patient listened to and sang along with songs that held personal emotional value—specifically, “Acqua Azzurra” and “La Rondine”. These songs were selected in consultation with the patient’s family, based on her autobiographical memories and preferences, as assessed through the Musical–Sound Anamnesis Form. This connection to meaningful music reduced anxiety and enhanced motivation and engagement in rehabilitation, transforming each session into a journey of self-expression and communication. The chosen songs were also used during fMRI acquisition to align therapeutic and neuroimaging contexts.

The TS was delivered by a certified Neurologic Music Therapist and followed the principles of the NMT model. No separate vocal warm-up was provided, as the MIT component preceding each TS session had already activated the vocal function. As per standard NMT clinical practice, during TS, the therapist guided the patient through active singing, with attention to expressive prosody, articulation, and respiratory control. The therapeutic strategies included the repetition of selected lines, cueing, pacing adjustments, and verbal–emotional facilitation.

The division of the 4 -min session into 25 min of MIT followed by 20 min of TS was based on the clinical judgment and expertise of the NMT-certified therapist. Starting with MIT allowed for structured language activation through hierarchical repetition and rhythmic pacing, while concluding with TS promoted emotional engagement, self-expression, and positive affect. This structure was also designed to end each session on a motivating and emotionally rewarding note, enhancing the patient’s adherence and the overall rehabilitation experience.

All Neurologic Music Therapy sessions (MIT and TS) were delivered in a dedicated therapy room within the rehabilitation ward, designed to minimize distractions and ensure adequate acoustic conditions. Throughout the program, the patient’s engagement was high, facilitated by the emotional salience of the selected material and the consistency of the therapeutic environment. The MIT training is shown in Table 1.

In addition to the NMT-based intervention, the patient received standard speech–language therapy as part of her inpatient rehabilitation program. These sessions were conducted three times per week by a speech–language therapist and focused on traditional language rehabilitation techniques. These included oral motor training, phonemic cueing, repetition exercises, and confrontation naming tasks, following a cognitive–linguistic therapy model. The conventional therapy was delivered in parallel with the MIT and TS sessions but in separate sessions, and it was not based on musical or rhythmic stimulation.

### 2.4. MRI Examination

This study complies with recommended standards for fMRI reporting, and detailed acquisition and analysis parameters are described below, following guidelines from the Committee on Best Practices in Data Analysis and Sharing (COBIDAS).

The patient underwent an MRI examination with an MRI scanner rating at 3.0 T (Achieva, Philips Healthcare, Best, The Netherlands), using a 32-channel phased-array RF head coil. The protocol included T1-weighted 3D Turbo Field Echo (sT1W-3D-TFE) images [TR = 8 ms, TE = 4 ms, slice thickness/gap = 1/0 mm, number of slices= 185], used as the structural reference for the subsequent functional analysis.

Functional MRI data were acquired using a T2-weighted gradient-echo echo-planar imaging (EPI) sequence [TR = 2000 ms, TE = 30 ms, flip angle = standard, slice thickness = 4 mm, 31 axial slices, in-plane resolution = 2 × 2 mm, voxel size of 2 × 2 × 4 mm^3^] oriented axially to cover the entire brain. To ensure a high signal-to-noise ratio and minimize scanner interference with auditory stimuli, noise-attenuating headphones were used, and stimuli were preprocessed to enhance the signal clarity. The auditory tasks were synchronized with the scan sequences to avoid peak acoustic noise, and a regressor for scanner noise was included in the design matrix to isolate the BOLD signal specific to the auditory tasks.

### 2.5. fMRI Experiment

During both T0 and T1 sessions, fMRI runs were conducted using a block paradigm that alternated between three 40 s task periods and three 20 s rest periods. The tasks involved listening to four different songs, “Acqua Azzurra”, “La Rondine”, the neutral “Azzurro”, and “Malafemmina”, each repeated twice in the sequence. We implemented several strategies to minimize the interference caused by the scanner noise and to isolate the brain’s response to the auditory stimuli. To reduce the impact of background noise, we used noise-attenuating headphones that effectively suppressed the scanner noise, while ensuring that the auditory stimuli were presented clearly to the participant. Additionally, the auditory stimuli were preprocessed to optimize their signal-to-noise ratio, making them more distinguishable against any residual background noise. Moreover, the presentation of auditory stimuli was carefully synchronized with the MRI scanning sequence to avoid periods of peak acoustic noise. This synchronization helped to reduce potential interference during the acquisition of functional data. In the analysis phase, we further accounted for any residual effects of scanner noise by including a regressor in the design matrix to capture its temporal pattern. These measures were taken to ensure that the recorded neural activations primarily reflected the brain’s response to the auditory stimuli and not to the scanner noise.

### 2.6. fMRI Analysis

Functional imaging data were analyzed using FSL (FMRIB’s Software Library, version 6.0.6, https://fsl.fmrib.ox.ac.uk/fsl/docs/#/ accessed on 10 February 2025) in accordance with current best practices for fMRI analysis. The preprocessing steps included motion correction through MCFLIRT, following by the removal of non-brain tissue using BET, and spatial smoothing with a Gaussian kernel of a 6 mm full width at half-maximum (FWHM) [46]. A mean-based intensity normalization of all volumes was performed, along with a high-pass temporal filter with a sigma value of 30 s to remove low-frequency drifts (sigma = 30 s).

Lesions were manually segmented using ITK-SNAP, and a binary lesion mask was created to exclude damaged brain tissue from the analyses. Functional images were co-registered to the individual’s T1-weighted structural scan and normalized to the MNI 2 mm template using non-linear registration procedures optimized for lesioned brains. Functional images were aligned to high-resolution structural images using FLIRT [47,48].

The fMRI task design followed a block paradigm, consisting of alternating three 40 s task periods—where the patient listened to emotionally meaningful or neutral musical stimuli—and three 20 s rest periods. The design matrix included the time course of the music listening task as the main explanatory variable, convolved with a Double-Gamma Hemodynamic Response Function, which included a canonical positive gamma function and a delayed undershoot component to model the BOLD response accurately. To identify significant brain activations, cluster-based inference was performed using a Z-score threshold of 2.3 and a family-wise error (FWE) corrected cluster significance threshold of *p* < 0.05. Activation maps were visually inspected and interpreted with anatomical reference to the Harvard–Oxford cortical atlas, ensuring a robust and anatomically meaningful localization of the functional data.

### 2.7. Statistical Analysis

To assess the significance of the changes observed between pre- and post-treatment scores in a single patient undergoing our protocol, the following analyses were performed: reliable change index (RCI), Cohen’s d (Effect Size), and percentage change.

The RCI was calculated to determine whether the observed changes between pre- and post-treatment scores were statistically reliable and not attributable to measurement error. The RCI formula is

$$RCI=\frac{Post\text{-}Treatment\;Score-Pre\text{-}Treatment\;Score}{Standard\;Error\;of\;Measurement\;(SEM)}$$

For this analysis, a hypothetical Standard Error of Measurement (SEM) of 5 was assumed, as specific reliability data for each test were not available. The RCI values were then compared to the threshold of 1.96, which indicates a statistically reliable change at the 95% confidence level (RCI > 1.96: statistically reliable improvement; RCI < −1.96: statistically reliable decline; RCI between −1.96 and 1.96: no statistically significant change).

To quantify the magnitude of the changes between pre- and post-treatment scores, the Cohen’s d was calculated for each test. Cohen’s d helps measure the effect size, providing insight into the practical significance of the observed changes. The formula used is

$$Cohen^{’}s\;d=\frac{Mean\;of\;Post\text{-}Treatment\;Scores-Mean\;of\;Pre\text{-}Treatment\;Scores}{Standard\;Deviation\;of\;Differeces}$$

A standard deviation of 5 was assumed, and the effect sizes were interpreted as follows:

d ≈ 0.2: small effect; d ≈ 0.5: medium effect; d ≈ 0.8 or higher: large effect.

To provide a relative comparison of the improvement in each measure, the percentage change was calculated using the formula

$$Percentage\;Change=\frac{Post\text{-}Treatment\;Score-Pre\text{-}Treatment\;Score}{Pre\text{-}treatment\;Score}\;\times \;100$$

The results were analyzed using single-case statistical methods. Pre- and post-intervention scores were evaluated through the RCI, Cohen’s d, and percentage change to determine their clinical and statistical significance. These methods are consistent with recommendations for single-subject experimental designs and adhere to the SCRIBE 2016 guidelines for behavioral interventions.

## 3. Results

### 3.1. Neuropsychological Results

**Figure 1:**
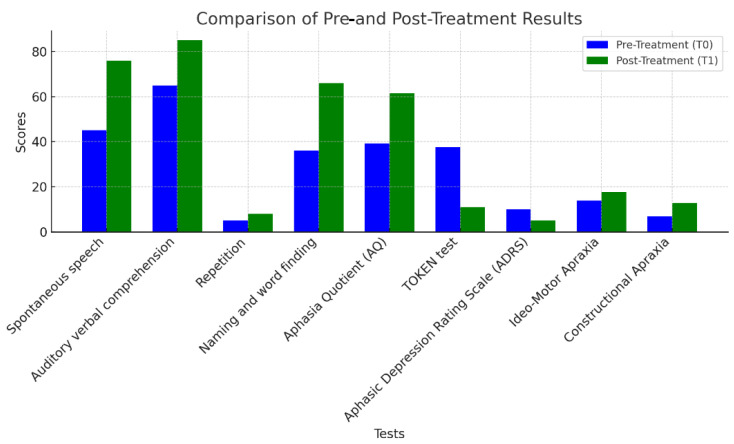
Patient’s pre- and post-intervention performance across key cognitive and language domains. The graphical representation was designed to facilitate the visual comparison of standardized scores, highlighting the changes following the NMT intervention. The blue bars represent the pre-treatment scores, while the green bars show the post-treatment scores, making it easy to see the improvements across different measures.

The MIT training led to substantial improvements across all neuropsychological assessments. Notable increases were observed in spontaneous speech (RCI = 6.20, 68.9% increase), verbal comprehension (RCI = 4.00, Cohen’s d = 4.00, 30.8% increase), word repetition (60% increase), and naming (83.3% increase, Cohen’s d = 6.00). The overall AQ improved from 39.3 to 61.4 (RCI = 4.42, 56.2% increase), indicating a shift from severe to moderate aphasia. Additionally, the Token Test scores improved significantly, demonstrating enhanced comprehension and task performance (70.7% improvement). Emotional well-being also showed progress, with a 50% reduction in depressive symptoms as measured by the ADRS. Motor skills improvements included a 29.1% increase in ideomotor ability and an 88.9% increase in constructional apraxia, highlighting recovery in fine motor skills. These improvements are clearly shown in Table 2 and Figure 1.

### 3.2. fMRI Results

Brain activation clusters were anatomically localized using the Harvard–Oxford cortical and subcortical structural atlases, aligned to the MNI 152 standard space. All reported peak activations refer to MNI coordinates and were thresholded at Z > 2.3 with FWE-corrected *p* < 0.05.

*General Neural Activations During Tasks vs. Rest (T0 and T1):*

**Figure 2:**
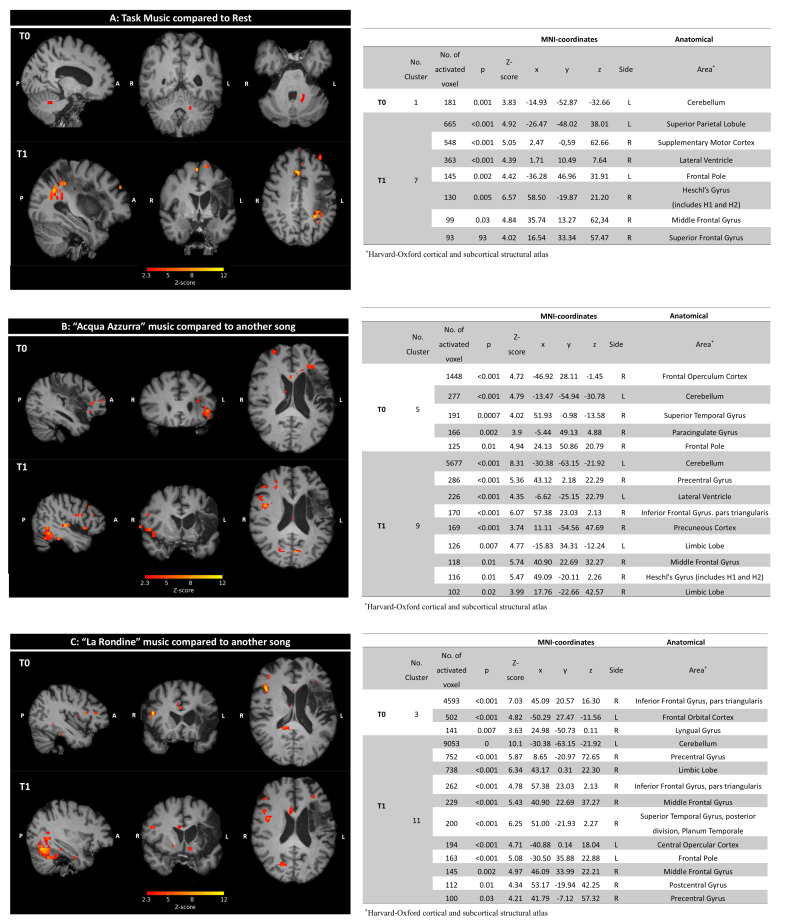
On the left side. (**A**): Task music compared to rest: brain activation maps during task music compared to rest at baseline (T0) and after treatment (T1). The highlighted clusters indicate areas with significant activation differences between the two conditions at each time point. (**B**): “Acqua Azzurra” compared to another song: brain activation maps for listening to “Acqua Azzurra” compared to another song at baseline (T0) and after treatment (T1). The highlighted clusters indicate areas with significant activation differences between the two conditions at each time point. (**C**): “La Rondine” compared to another song: brain activation maps for listening to “La Rondine” compared to another song at baseline (T0) and after treatment (T1). The highlighted clusters indicate areas with significant activation differences between the two conditions at each time point. On the right side. Statistical data, MNI coordinates (x, y, z), and the respective anatomical areas obtained from the task at T0 and T1. Only the details of the anatomical areas of the main cluster are tabulated.

At the initial scan (T0), significant activation was observed in the left cerebellum (181 voxels, Z-score = 3.83, *p* = 0.001), illustrating robust engagement during music listening tasks. By the follow-up (T1), the data showed an increase in both the number and intensity of activated clusters. Notable areas of activation included the right hemisphere’s supplementary motor cortex (Z-score = 5.05, *p* < 0.001), lateral ventricle (Z-score = 4.39, *p* < 0.001), and superior frontal gyrus (Z-score = 4.02, *p* = 0.04), along with activations in the left hemisphere’s superior parietal lobule (Z-score = 4.92, *p* < 0.001) and frontal pole (Z-score = 4.42, *p* = 0.002) (see Figure 2A-T1 for enhanced activations).

*Song-Specific Neural Activations (“Acqua Azzurra” vs. Other Songs):*

Initial activations at T0 for “Acqua Azzurra” were concentrated in clusters on the right hemisphere, including the frontal operculum cortex (Z-score = 4.72, *p* < 0.001) and superior temporal gyrus (Z-score = 4.02, *p* = 0.0007). By T1, there was a noticeable increase in activated regions, with significant new activations in the right hemisphere’s precentral gyrus (Z-score = 5.36, *p* < 0.001), inferior frontal gyrus (Z-score = 6.07, *p* < 0.001), and middle frontal cortex (Z-score = 5.74, *p* = 0.01). The left cerebellum also showed a significant activity increase (Z-score = 8.31, *p* < 0.001).

*Song-Specific Neural Activations (“La Rondine “ vs. Other Songs):*

At T0, “La Rondine” induced fewer activations, with significant findings in the right inferior frontal gyrus (Z-score = 7.03, *p* < 0.001) and left frontal orbital cortex (Z-score = 4.82, *p* < 0.001). By T1, the extent of neural activations expanded dramatically, involving eleven clusters, with notable activations in areas such as the right precentral gyrus (Z-score = 5.87, *p* < 0.001), limbic lobe (Z-score = 6.34, *p* < 0.001), and left cerebellum (Z-score = 10.1, *p* < 0.01).

Visual illustrations of these changes are detailed in Figure 2A–C for each corresponding music task and rest comparison.

## 4. Discussion

This case report substantiates the efficacy of NMT in the neurorehabilitation of PSA, with significant enhancements in language and communicative abilities corroborated by neuroplastic changes observed via fMRI.

To our knowledge, this is the first documented case combining MIT and TS in the rehabilitation of PSA, supported by both pre- and post-intervention neuropsychological assessment and fMRI data. While MIT is well-established in the literature, TS has not been systematically studied in conjunction with MIT nor in association with objective neurofunctional outcomes.

In our study, neuropsychological assessment showed clinically relevant improvements in both language and cognitive domains. The patient exhibited gains in naming, phonological access, and sentence repetition (core deficits in non-fluent aphasia), alongside improvements in attention and working memory. These changes suggest that the intervention had an effect beyond rote language learning, potentially enhancing the underlying executive functions that support communication. These findings are consistent with prior studies demonstrating that MIT can promote naming, repetition, and articulatory fluency in patients with chronic non-fluent aphasia [27,34]. Moreover, in line with our results, the literature highlights the potential of rhythmic and melodic stimulation to improve executive functions such as attention and working memory [49], and to support neuroplastic changes related to language recovery [37,50].

Notably, improvements in linguistic performance were paralleled by a functional reorganization in brain activation patterns. Firstly, the key findings reveal increased activation in peri-lesional left-hemisphere areas, notably the superior frontal gyrus and superior parietal lobule, suggesting that MIT stimulates these areas through its rhythmic and melodic components, thereby enhancing their connectivity with language-related regions [51,52,53]. Secondly, the data indicate that MIT supports compensatory neural reorganization within the right hemisphere, enhancing language production and auditory processing capabilities [20,24,37,54,55,56,57]. The integration of TS may have emphasized MIT’s pivotal role in activating right-hemisphere mechanisms essential for language recovery [38,58,59]. In fact, emotional and autobiographically significant music, as used in TS, likely further enhanced engagement and motivation, supporting cognitive activation through limbic–frontal integration [39]. The combined use of MIT and TS thus appears to stimulate both linguistic and cognitive networks through rhythmic–melodic entrainment, multisensory integration, and emotionally meaningful engagement. Significant cerebellar involvement was noted, with the left cerebellum showing extensive activation during tasks, underscoring its role in the melodic and rhythmic integration critical for speech production. This suggests the cerebellum’s broader contribution to cognitive functions like sequencing and synchronization necessary for language processing, which is enhanced during musical activities, promoting emotional and motivational engagement [39,40]. Our analysis also demonstrated robust neural activation in critical areas such as the superior frontal gyrus and the left superior parietal lobule during musical tasks, confirming that music listening and singing extend beyond the simple engagement of neural circuits to actively promoting motor–auditory integration, crucial for MIT’s efficacy [27,37,38,50,51,58,59]. The distinct structure of NMT language techniques, as opposed to the broader scope of traditional Music Therapy that incorporates a wider array of musical activities, suggests a complementary role in neurorehabilitation, enhancing cognitive and emotional outcomes through different musical interventions [24,25,27,32,39,49,57].

A further strength of this study is that the intervention was tailored to the patient’s clinical and personal profile. While she was receiving conventional speech–language therapy three times per week (focused on standard techniques such as naming, repetition, and phonemic cueing), she was also undergoing NMT sessions three times per week. This structure reflects real-world multidisciplinary practice in a Research Hospital. The NMT therapist structured the 45 min sessions into 25 min of MIT followed by 20 min of TS. This structure was not arbitrary but based on clinical reasoning: MIT, being cognitively demanding, was placed first to optimize performance, while TS, being emotionally engaging and prosodically expressive, was used to consolidate progress and end the session on a positive note.

MIT sessions typically focused on one phrase per session, progressing through the protocol steps as described in the Supplementary Materials File. The number of phrases addressed varied according to the patient’s daily performance, attention, and fatigue level, factors common in neurorehabilitation. Previously mastered phrases were briefly reviewed at the beginning of each session. This adaptive approach ensured clinical feasibility and therapeutic rigor.

Acknowledging the limitations of the single-case design and the absence of language specific tasks in the fMRI protocol, future research should integrate linguistically focused neuroimaging tasks to more accurately assess the relationship between neural activation and language improvement. Additionally, expanding the sample size and demographic diversity would help establish MIT’s efficacy and generalize these findings across broader populations.

## 5. Conclusions

Although causality cannot be established in a single case, the convergence of behavioral and neural data reinforces the potential efficacy of NMT techniques as effective adjunctive tools in PSA rehabilitation. When delivered within a structured and personalized framework, NMT appears to facilitate functional language recovery and induce measurable neuroplastic changes, especially when integrated with emotionally meaningful musical elements.

## Figures and Tables

**Table 1:** Melodic Intonation Therapy (MIT) procedure.

MIT	Step	Methodology
LEVEL 1	1 Humming	The therapist begins by displaying a visual cue and then vocalizes the target phrase once, humming at a pace of 1 syllable/sec. Following this, the therapist sings the phrase twice while tapping the patient’s left hand once per syllable
	2 Unison Intoning	The therapist and patient sing the target phrase together while the therapist taps the patient’s left hand once per syllable
	3 Unison Intoning with Fading	The therapist and patient start by singing and tapping the target phrase together. Halfway through, the therapist gradually stops, allowing the patient to finish singing the rest of the phrase with hand-tapping but without any additional verbal or facial cues
	4 Immediate Repetition	The therapist sings and taps the target phrase while the patient listens. Immediately afterward, the patient repeats the phrase, aided only by tapping on the left hand
	5 Response to a Probe Question	Immediately after the patient successfully repeats the target phrase (step 4), the therapist quickly asks a question, such as “What did you say?” and the patient then responds by singing the target phrase. Hand-tapping is the only form of assistance permitted
LEVEL 2	1 Target Phrase Introduction	The therapist presents a visual cue and intones the phrase twice, maintaining a rate of 1 syllable/second, while tapping the patient’s left hand once for each syllable
	2 Unison with Fading	The therapist and patient start by singing and tapping the target phrase together. Halfway through, the therapist gradually stops, allowing the patient to finish singing the rest of the phrase with hand-tapping but without any additional verbal or facial cues
	3 Delayed Repetition	The therapist intones and taps the target phrase while the patient listens. After a 6 s delay, the patient repeats the phrase, assisted only by tapping on the left hand. No verbal assistance is provided
	4 Response to a Probe Question	After the patient successfully repeats the target phrase (step 3), the therapist waits for 6 s. Then, the therapist intones a question about the target phrase, and the patient answers by intoning the target phrase. No assistance is provided
LEVEL 3	1 Delayed repetition	The therapist intones and taps the target phrase while the patient listens. After a 6 s delay, the patient repeats the phrase, assisted only by tapping on the left hand. No verbal assistance is provided
	2 Introducing Sprechgesang	The therapist presents the target phrase in sprechgesang (a style combining speech and song) twice, accompanied by hand-tapping, while the patient listens. The words are delivered slowly, with an exaggerated emphasis on rhythm and stressed (accented) syllables, rather than being sung
	3 Sprechgesang with Fading	The therapist and patient start by singing and tapping the target phrase together. Halfway through, the therapist fades out, allowing the patient to finish the rest of the phrase
	4 Delayed Spoken Repetition	The therapist presents the target phrase using normal speech prosody, without hand-tapping, while the patient listens. After a 6 s delay, the patient repeats the phrase using normal speech
	5 Response to a Probe Question	After a 6 s delay, the therapist asks a question to prompt the patient to produce the target phrase using normal speech. The patient responds by speaking the target phrase without any assistance

**Table 2:** Neuropsychological battery administered before training (T0) and after training (t1).

Test	Pre-Treatment (T0)	Post-Treatment (T1)	RCI	Difference	Cohen’s d	Percentage Change (%)
**Spontaneous speech**	45	76	6.2	31	6.2	68.88
**Auditory verbal** **comprehension**	65	85	4	20	4	30.77
**Repetition**	5	8	0.6	3	0.6	60
**Naming and word finding**	36	66	6	30	6	83.33
**Aphasia Quotient (AQ)**	39.3	61.4	4.42	22.1	4.42	56.23
**Token Test**	37.5	11	−5.3	−26.5	−5.3	−70.66
**Aphasic Depression Rating Scale (ADRS)**	10	5	−1	−5	−1	−50
**Ideomotor apraxia**	13.75	17.75	0.8	4	0.8	29.09
**Constructional apraxia**	6.75	12.75	1.2	6	1.2	88.88

## Data Availability

The authors will provide the raw data supporting the conclusions of this article upon request, without unnecessary restrictions.

## Supplementary Materials

The following supporting information can be downloaded at: https://www.mdpi.com/article/10.3390/jcm14103436/s1, Supplementary Materials File.
