# Supplementary material for: The Use of Neurologic Music Therapy in Post-Stroke Aphasia Recovery: A Case Report on Linguistic Improvements and fMRI Correlates
Source: J Clin Med. 2025 May 14;14(10):3436. doi: 10.3390/jcm14103436 (PMC12112711; doi:10.3390/jcm14103436)
Supplement: Supplementary file 1 [file jcm-14-03436-s001.zip › jcm-3582564-supplementary.pdf]

Melodic Intonation Therapy (MIT)

Structure of the Intervention

The MIT intervention was based on a four-level hierarchical protocol adapted for Italian-speaking patients with non-fluent aphasia. Each level consisted of 4–5 steps, progressing from simple unison repetition with rhythmic tapping to autonomous verbal production with natural prosody. The therapist scored the patient’s performance at each step to guide progression and ensure structured delivery.

	STIMULUS	ANSWER	CONSEQUENCE	SCORE
FIRST LEVEL				
	The therapist sings the melody 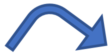 With a rhithmic tapping on the patient’s left hand (x2)	The patient sings the melody in unison with the therapist, continuous tapping (x2)	If correct, proceed to the next level	
SECOND LEVEL				
Step 1	Inform the patient not to repeat; present the melody and the word once 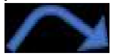 C I A O	The therapist presents the stimulus and moves the patient’s hand rhythmically together with them.	Proceed to Step 2	
Step 2	The therapist presents the item, then invites the patient to repeat it together with him	Therapist and patient sing in unison while moving the hand rhythmically.	If the patient performs correctly, assign one point and proceed to Step 3. If not correct, assign zero and return to Step 1 with a different melodic pattern.	
Step 3	The therapist sings the item twice and asks the patient to join in.	Patient and therapist begin the response in unison, with the therapist gradually fading out. Continuous tapping is maintained.	If the patient performs correctly, assign one point and proceed to Step 4. If not correct, assign zero and return to Step 1 with a different melodic pattern.	
Step4	The therapist asks the patient to listen and sings the item twice, then asks her/him to repeat it. Continuous tapping is maintained.	The patient repeats the item, and tapping continues.	If the patient performs correctly, assign one point and proceed to Step 5. If not correct, assign zero and return to Step 1 with a different melodic pattern.	
Step 5	The therapist sings a phrase or question containing the target word.	The patient repeats the item accompanied by tapping from the therapist.	If the patient performs correctly, assign one point. If not correct, assign zero and start again	

THIRD LEVEL				
Step 1	Inform the patient not to repeat and present the item twice.	While presenting the item, the therapist supports the patient's tapping.	Proceed to Step 2.	
Step 2	The therapist presents the item and invites the patient to participate	Therapist and patient initiate the response together	If the patient performs correctly, assign one point and proceed to Step 3. If not correct, assign zero and return to Step 1 with a different melodic pattern	
Step 3	The therapist asks the patient to listen and sings the item twice, then asks the patient to repeat it.	The patient repeats the item while continuing hand movement together with the therapist.	If the patient performs correctly, assign 2 points and proceed to Step 4. If not correct, go back to Step 2 and retry Step 3. If successful on the retry, assign 1 point. If not correct again, stop the procedure and return to Step 1 with a new item	
Step4	The therapist sings a question containing parts of the target item.	The patient responds by singing the item.	If correct, assign 2 points. If not correct, return to Step 3 and retry Step 4; if successful on the retry, assign 1 point. If not correct again, stop the procedure.	
FOURTH LEVEL				
Step 1	The therapist asks the patient to listen and sings the item twice, then asks her/him to repeat it	The patient repeats the item while continuing hand movement together with the therapist	If the patient performs correctly, assign 2 points and proceed to Step 2. If not correct, return to Step 2 of Level Three and retry; if successful, assign 1 point. If still incorrect, stop the procedure and return to Step 1 with a new item.	
Step 2	The therapist presents the item using sprechgesang and invites the patient to join in (twice).	The patient sings the response in sprechgesang in unison, while the therapist's voice fades out progressively, maintaining rhythmic tapping.	If the patient performs correctly, assign 2 points and proceed to Step 3. If not correct, return to Step 1. If correct on retry, assign 1 point. If incorrect again, stop the procedure and return to Step 1 with a new item.	
Step 3	The therapist asks the patient to listen and presents the item in sprechgesang with hand tapping, then asks the patient to repeat it.	The patient repeats the item in sprechgesang without hand tapping	If the patient performs correctly, assign 2 points and proceed to Step 4. If not correct, return to Step 2 and retry Step 3. If successful, assign 1 point. If still incorrect, stop the procedure and return to Step 1 with a new item.	
Step4	The therapist asks the patient to listen and presents the item with natural prosody, then asks the patient to repeat it. No tapping is used.	The patient repeats the item with natural prosody and without hand tapping.	If the patient performs correctly, assign 2 points and proceed to Step 5. If not correct, return to Step 3 and retry Step 4. If correct on retry, assign 1 point. If still incorrect, stop the procedure and return to Step 1 with a new item	
Step 5	The therapist asks one or more questions that contain parts of or the entire target item.	The patient responds appropriately and accurately.	If correct, assign 2 points. If not correct, return to Step 4 and retry Step 5. If correct on retry, assign 1 point. If still incorrect, stop the procedure.	
			**TOTAL**	

Sample of High-Frequency and Functional Phrases Used

**Phrase**	**English Equivalent**
Mi chiamo…	My name is…
Come stai?	How are you?
Sto bene / Sto male	I’m fine / I’m not well
Ho fame / Ho sonno	I’m hungry / I’m sleepy
Ti voglio bene	I love you (affectionately)
Buongiorno / Buonanotte	Good morning / Good night
Accendi la luce	Turn on the light
Devo andare in bagno	I need to go to the bathroom
Andiamo in palestra	Let’s go to the gym
Cantiamo?	Shall we sing?

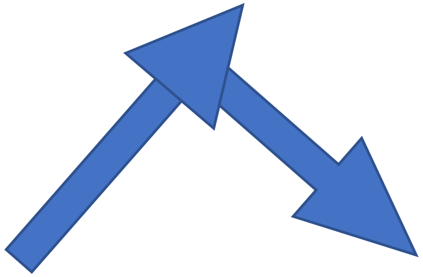

CI A O

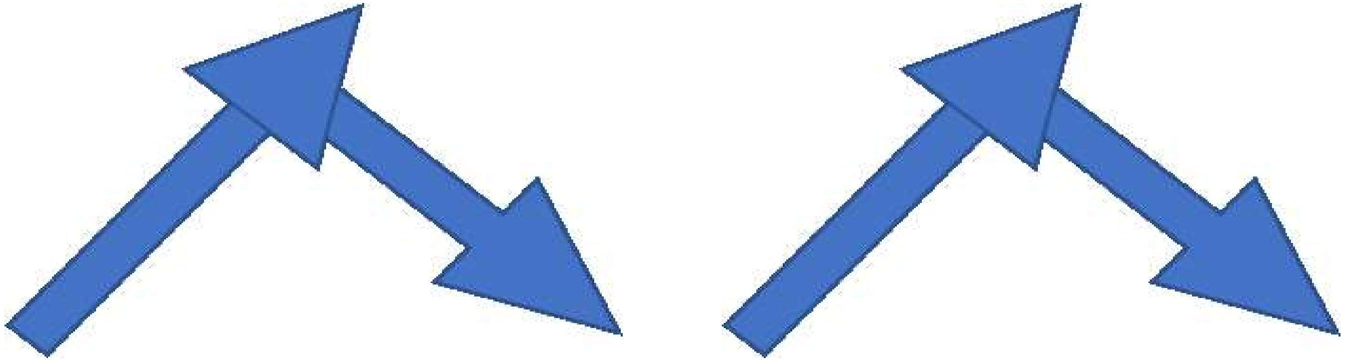

MI CHIA MO ....

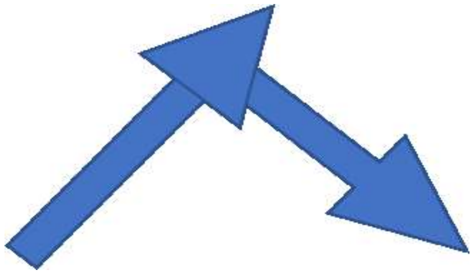 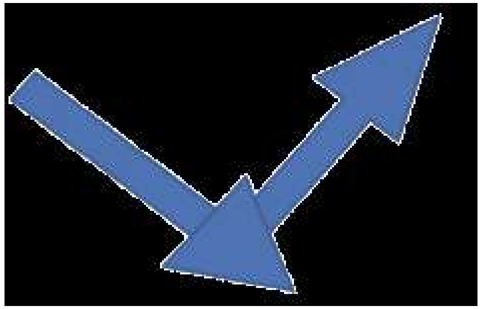

CO ME STA I ?

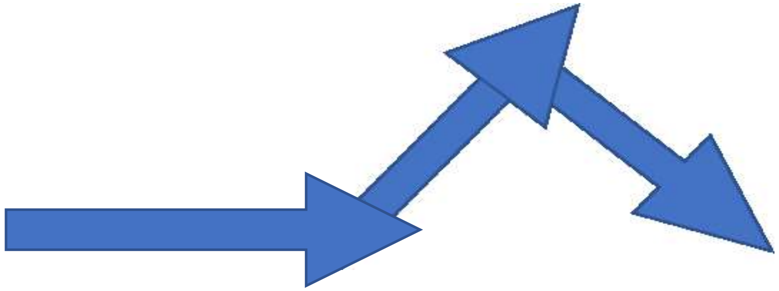

STO BE NE

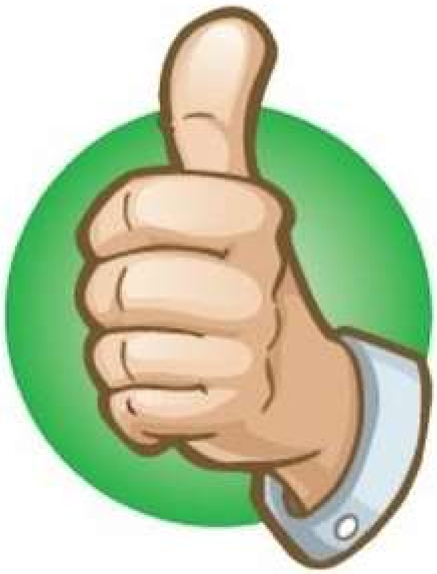

STO MA LE

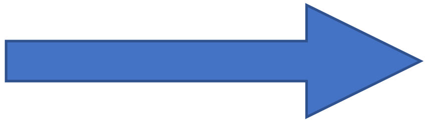

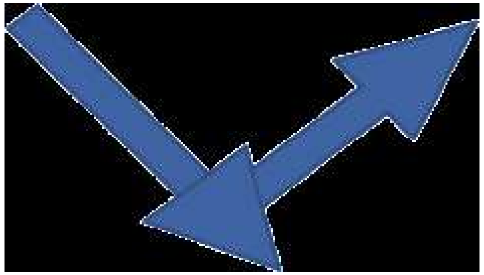

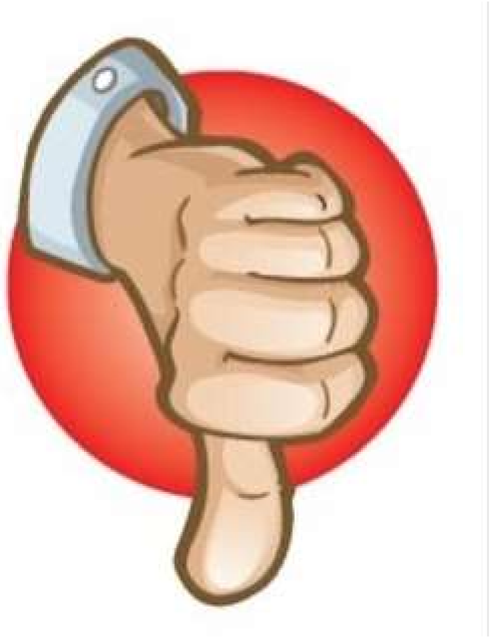

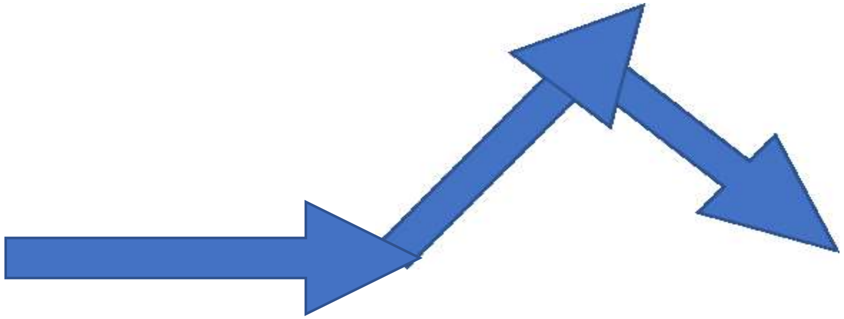

HO FA ME

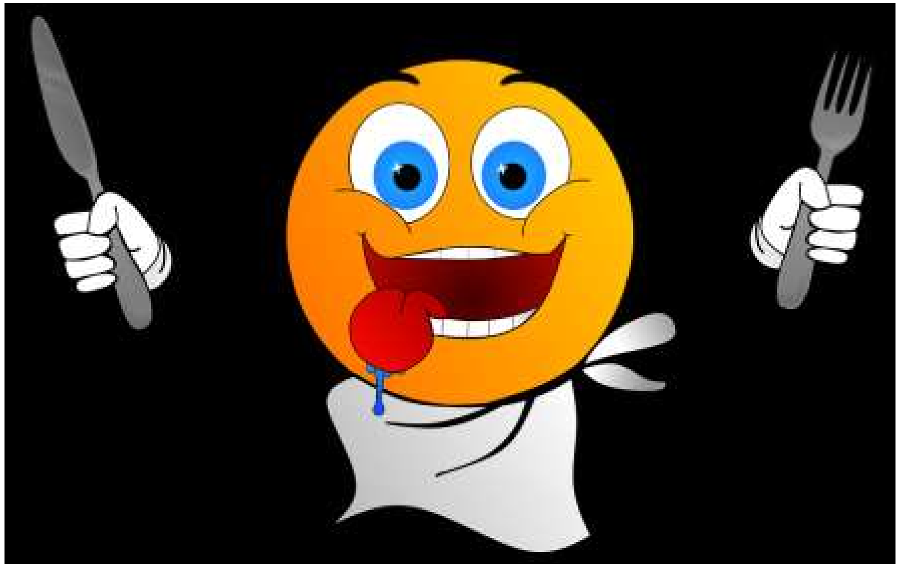

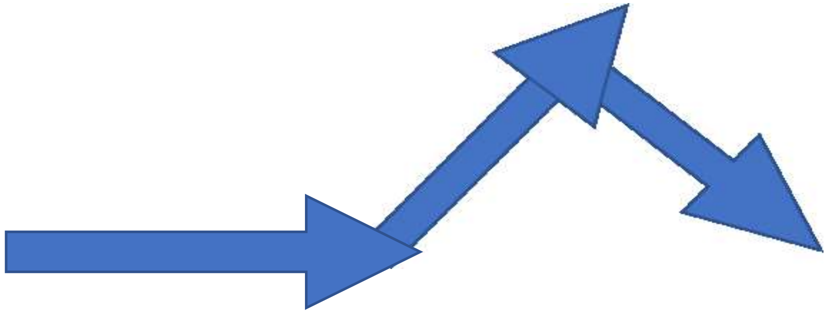

HO SE TE

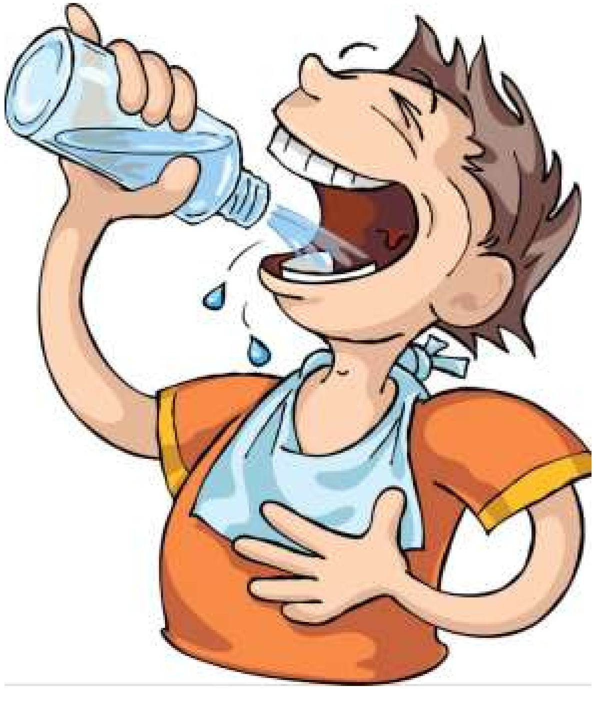

S I

N O

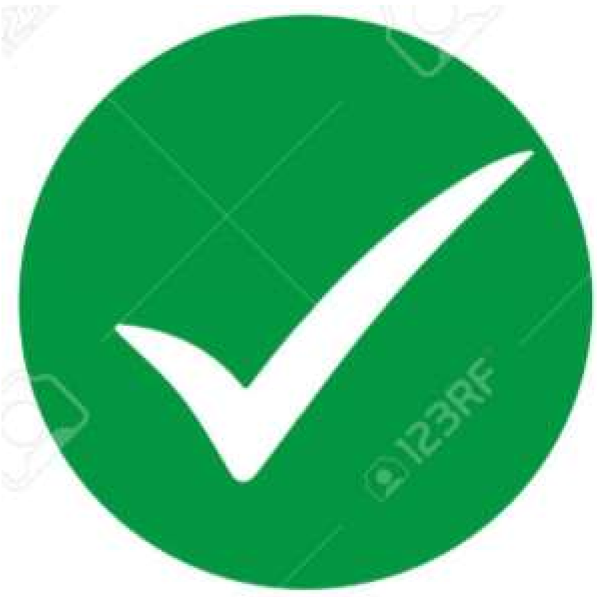

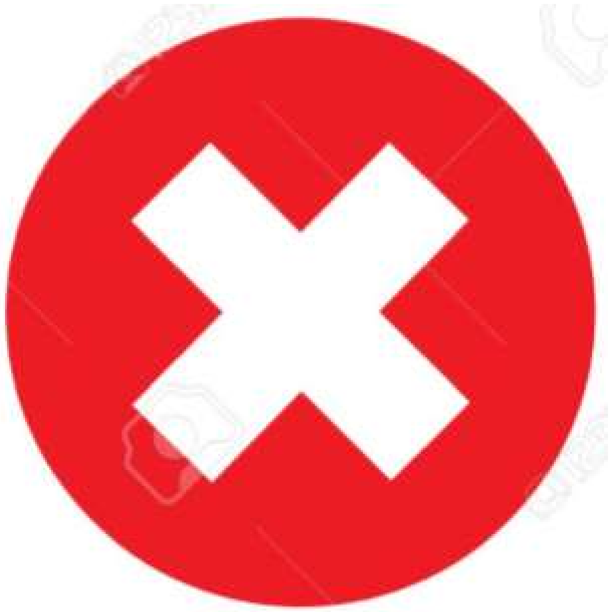

SCORING SHEET

This table illustrates the scoring method used by the therapist to monitor progress through the steps and levels of MIT. Each row corresponds to one target phrase, and points are assigned based on accuracy at each step.

NOME E COGNOME:

DATA:

ITEM	1° LIV.	2° LIVELLO					3° LIVELLO				4° LIVELLO					TOT
		STEP 1	STEP 2	STEP 3	STEP 4	STEP 5	STEP 1	STEP 2	STEP 3	STEP 4	STEP 1	STEP 2	STEP 3	STEP 4	STEP 5	
